# Radio-frequency single electron transistors in physically defined silicon quantum dots with a sensitive phase response

**DOI:** 10.1038/s41598-021-85231-4

**Published:** 2021-03-12

**Authors:** Raisei Mizokuchi, Sinan Bugu, Masaru Hirayama, Jun Yoneda, Tetsuo Kodera

**Affiliations:** 1 Department of Electrical and Electronic Engineering, Tokyo Institute of Technology, Meguro, Tokyo 152-8552 Japan; 2 Tokyo Tech Academy for Super Smart Society, Tokyo Institute of Technology, Meguro, Tokyo 152-8552 Japan

**Keywords:** Quantum dots, Electrical and electronic engineering, Electronic devices

## Abstract

Radio-frequency reflectometry techniques are instrumental for spin qubit readout in semiconductor quantum dots. However, a large phase response is difficult to achieve in practice. In this work, we report radio-frequency single electron transistors using physically defined quantum dots in silicon-on-insulator. We study quantum dots which do not have the top gate structure considered to hinder radio frequency reflectometry measurements using physically defined quantum dots. Based on the model which properly takes into account the parasitic components, we precisely determine the gate-dependent device admittance. Clear Coulomb peaks are observed in the amplitude and the phase of the reflection coefficient, with a remarkably large phase signal of ∼45°. Electrical circuit analysis indicates that it can be attributed to a good impedance matching and a detuning from the resonance frequency. We anticipate that our results will be useful in designing and simulating reflectometry circuits to optimize qubit readout sensitivity and speed.

## Introduction

Spin qubits in silicon quantum dots (QD) are promising to realize a quantum computer, thanks to their small size and compatibility to the CMOS technology^1–6^. To adapt to the requirements for fast and quantum-non-demolition spin readout for fault-tolerant quantum computing^7–9^, radio-frequency (RF) reflectometry has been widely studied in the QD systems^10–16^. This technique utilizes impedance matching between the transmission line and the QD system within a resonator^17^. Therefore, modifications are often necessary to apply this technique to nanostructures with different designs. Recently, fast spin readout within coherence times was realized in a gate-defined silicon QD with a RF single electron transistor (RF-SET) charge sensor^10^.

Physically defined silicon QDs (PD-QDs) based on the silicon-on-insulator (SOI) technology^18–25^ would offer high flexibility in QD arrangement and suitability to dense two-dimensional QD integration without the need of gates for quantum confinement^22^. However, the PD-QD with the top gate structure is yet to be successfully combined with the RF-SET, possibly because of formation of an RC filter due to the reservoir of a resistive silicon channel and the capacitance of a 20-μm square top gate^10,11,26,27^. One way to avoid this problem is to simply reduce the size of the top gate^10,27^. An alternative solution would be to completely remove the top gate structure, made possible thanks to the SOI-based QD structures^22,28^. In this work, we report a large phase response and a detailed circuit analysis of RF-SET measurement in PD-QDs without the top gate structure.

The PD-QD devices were fabricated in line with our previous studies^22^. First, PD-QDs are formed by etching a 40-nm thick SOI layer. After thermal oxidation forming several nanometers of SiO_2_ on the SOI surface, phosphorus donors are implanted to the SOI layer except for a 20-μm square region around the QD and then activated by annealing in a nitrogen atmosphere. Finally, forming gas annealing is performed to terminate dangling bonds. In each device, the top gate structure is omitted to avoid RF leakage to the top gate; instead, the back gate is used to accumulate electrons.

## Results

### Device and measurement setup

**Figure 1:**
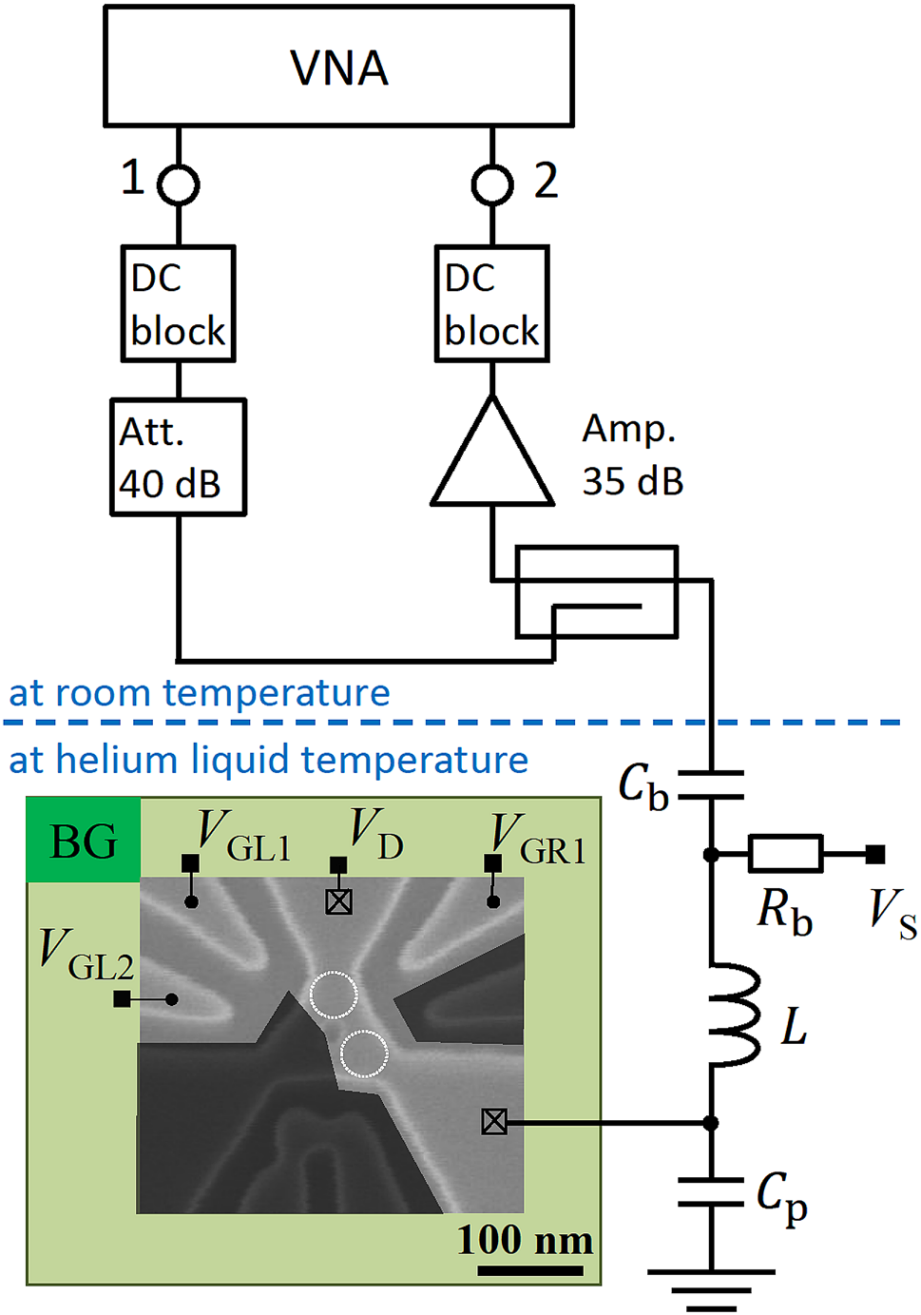
Measurement setup with a scanning electron micrograph of a physically defined quantum dot (PD-QD) device nominally identical to the measured ones. The PD-QDs do not have top gates. Drain voltage, $V_{{\text{D}}}$, source voltage, $V_{{\text{S}}}$, side gate voltages, $V_{{{\text{GL}}1}}$, $V_{{{\text{GL}}2}}$, and $V_{{{\text{GR}}1}}$, and back gate voltage, $V_{{{\text{BG}}}}$, can be applied. Shadowed areas indicate unused floating electrodes: a side gate, a single QD charge sensor, and a reservoir. An LC resonance circuit for impedance matching, which comprises a surface mount wire-wound inductor with an inductance $L$ = 680 nH and parasitic capacitance $C_{{\text{p}}} { }$, is connected to the source of the QD. In addition, an RC bias tee (*R*_b_ = 5 kΩ and $C_{{\text{b}}}$ = 4.7 μF) is connected to the LC circuit in series to apply a DC voltage $V_{{\text{S}}}$. For RF measurement, a vector network analyzer (VNA) outputs the RF signal from port 1, which is applied to the device after attenuation to suppress heating up the QD. The signal reflected from the device is branched by directional coupler and input to port 2 of VNA after amplification.

A scanning electron micrograph (SEM) of a PD-QD nominally identical to the ones used in this work is shown in Fig. 1. A charge sensor and side gates (SGs) are also formed in the same SOI layer as the QDs. However, some of them are set to be floating and do not play a role in the following experiments. A positive back gate voltage is applied to the Si substrate of the SOI wafer across the 145-nm thick buried oxide layer to accumulate electrons. The device is mounted on a printed circuit board (PCB) along with components for the bias tee and for the LC resonance circuit for RF-SET (Fig. 1). Parasitic capacitances of the PCB and the device form part of the LC circuit. All measurements in this paper are performed with devices immersed in liquid helium and hence at 4.2 K. We use a vector network analyzer (VNA) and apply the RF power output from port 1 of the VNA to the device after 60-dB attenuation. The signal reflected at the device is input into port 2 of the VNA after amplification of 35 dB. We note that the signal is amplified only at room temperature, not at cryogenic temperature.

### Frequency dependence and fitting

**Figure 2:**
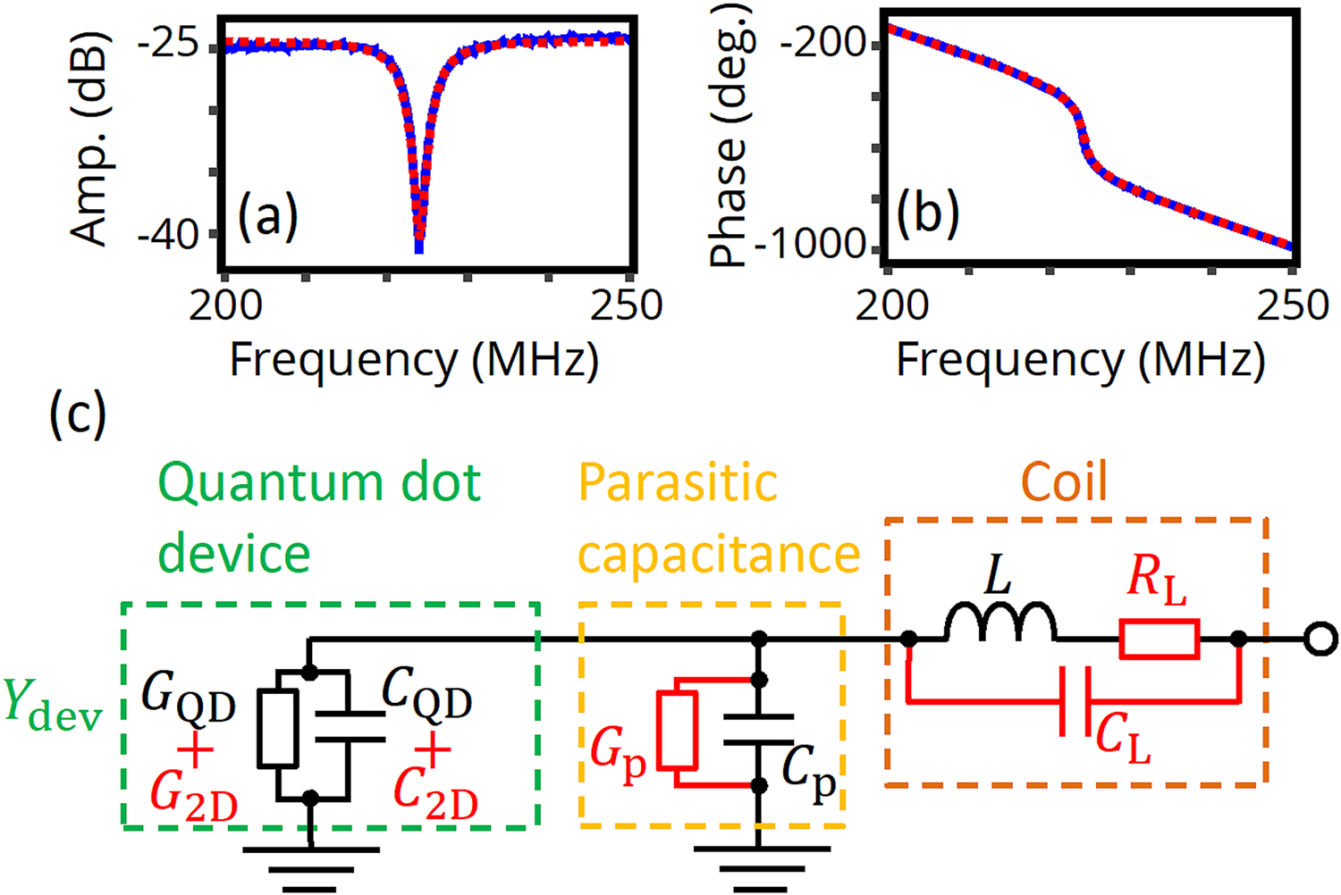
Frequency dependence of reflected RF signal and fitting using an equivalent circuit. (**a**,**b**) Amplitude and phase of the measured transmission signal from port 1 to port 2 at liquid helium temperature as a function of carrier frequency (blue solid lines). No DC voltages are applied to the device. Results from fitting based on the equivalent circuit in (**c**) are shown as well (red dotted lines). (**c**) An equivalent circuit for the load impedance, in which additional parasitic components are also taken into account.

First, we study the frequency dependence of the reflection coefficient at the device, ${\Gamma }$, without carrier accumulation using the back gate voltage $V_{{{\text{BG}}}}$ = 0 V (blue solid lines in Fig. 2a,b). Here, the charge sensor and all SGs are set to be floating for simplicity. A dip appears at a frequency which corresponds to the resonance of the circuit ($f_{r}$ = 224.063 MHz). In a simple description of the LC resonance circuit, the load impedance, $Z_{{{\text{load}}}}$, is proportional to device conductance at resonance; this would suggest that $Z_{{{\text{load}}}}$ should be close to zero in this case, which would result in almost complete reflection. This apparent discrepancy can be explained by the dielectric loss in the PCB which contributes as a conductance parallel to the device^12^. Given this, $Z_{{{\text{load}}}}$ has a finite value (at frequencies around the resonance) and ${\Gamma } = \left( {Z_{{{\text{load}}}} - Z_{0} } \right)/\left( {Z_{{{\text{load}}}} + Z_{0} } \right)$ can approach zero, where *Z*_0_ = 50 Ω is the characteristic impedance of the external signal line.

As we will see below, the frequency dependence of observed reflection can be described by the equivalent circuit shown in Fig. 2c. The circuit mainly consists of an inductance, $L$, a parasitic capacitance, $C_{{\text{p}}}$, and a QD impedance composed of a parallel circuit of a conductance $G_{{{\text{QD}}}}$ and a capacitance $C_{{{\text{QD}}}}$; however, each component has additional parasitic components in reality. Here, we take into account the parasitic capacitance and resistance in the coil, $C_{{\text{L}}}$ and $R_{{\text{L}}}$, and the parasitic conductance in the capacitor, $G_{{\text{p}}}$. $G_{{\text{p}}} { }$ and $R_{{\text{L}}}$ are due to dielectric loss and skin effect, respectively, so that each has a frequency dependence: $G_{{\text{p}}} = \omega C_{{\text{p}}} {\text{tan}}\delta$ and $R_{{\text{L}}} = \rho_{{\text{L}}} \sqrt \omega$, where $\omega$ = 2 $\pi f$ is the angular frequency, ${\text{tan}}\delta$ is loss tangent, and $\rho_{{\text{L}}}$ denotes a coefficient^29^. In addition, the effects of external components are considered, such as coaxial cables, attenuators, and amplifiers. Attenuation and amplification offset the amplitude of the reflected signal, and the coaxial cable causes a linear phase shift as a function of frequency due to its propagation constant.

Figure 2b,c show fitting results for the amplitude and the phase of the observed reflection at $V_{{{\text{BG}}}}$ = 0 V based on the equivalent circuit with following parameters (red dotted lines): $L$ = 680 nH, $\rho_{{\text{L}}}$ = 190 $\mu {\Omega }/\sqrt {{\text{rad}}/{\text{s}}}$, $C_{{\text{L}}} { }$ = 348 fF, $C_{{\text{p}}} { }$ = 394 fF, and ${\text{tan}}\delta$ = 0.00614. Here, to reduce the number of fitting parameters, the nominal value of $L$ is assumed, and $\rho_{{\text{L}}}$ is separately estimated from the measured transmission characteristic of an inductor nominally identical to the one used in these measurements (supporting information). The fittings have good agreements with the measurement results, proving the validity of the equivalent circuit model. The small deviation at off-resonance frequencies can be attributed to a background frequency dependence due to interference between the reflected signal and the isolation leakage in the directional coupler^29^. The necessity for additional parasitic components can be confirmed from fitting with a simple LCR circuit without taking into account additional parasitic components, where an inductance 3.5 times larger than $L$ is required for a good fitting (see supporting information for details).

### Back gate voltage dependence

**Figure 3:**
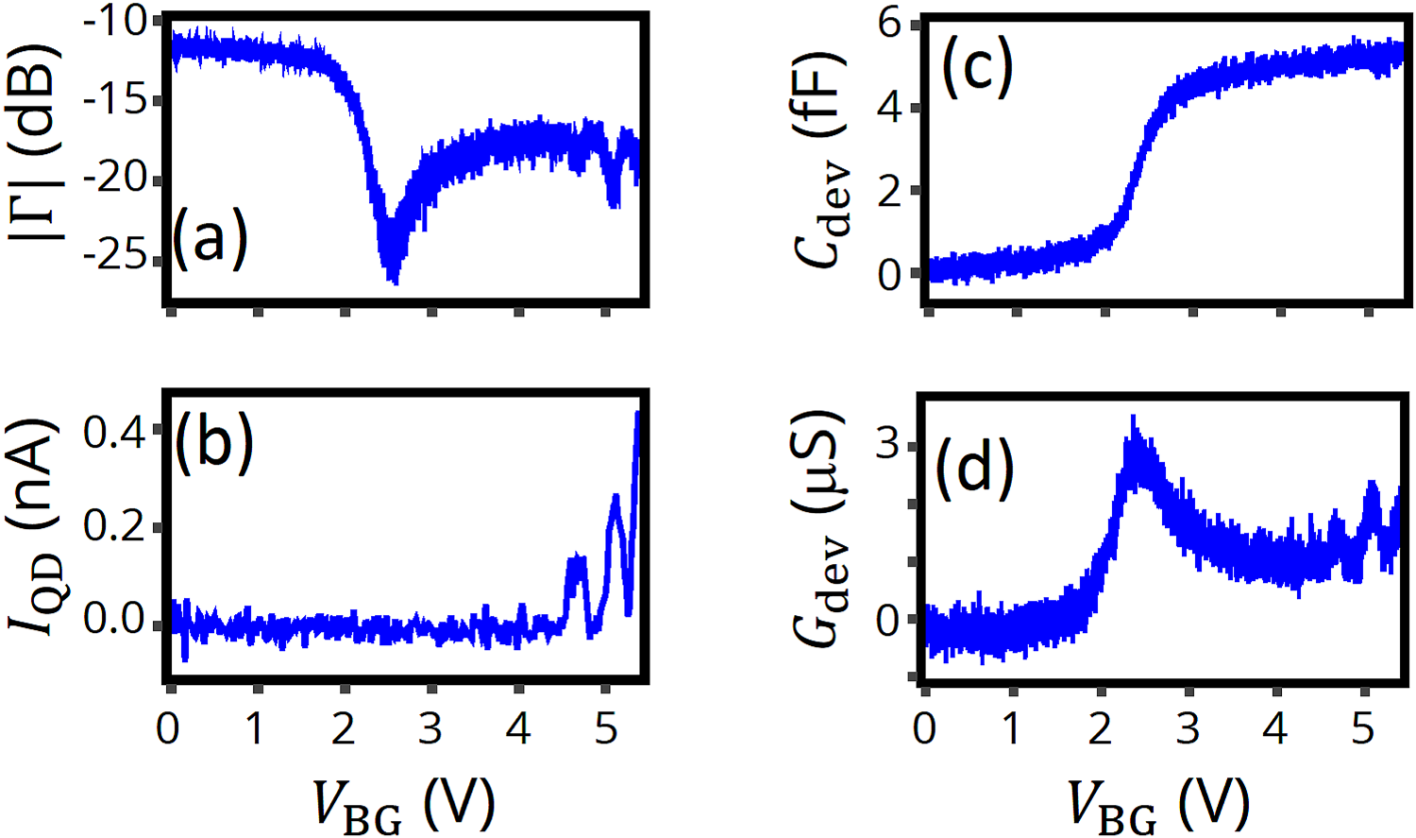
Back gate voltage dependence of RF reflectometry. (**a**,**b**) Amplitude of the reflection coefficient, $\left| {\Gamma } \right|$, and QD current, $I_{{{\text{QD}}}}$, as a function of $V_{{{\text{BG}}}}$ at liquid helium temperature. Carrier frequency $f$ = 223.464 MHz, $V_{{{\text{DS}}}}$ = 5 mV. $V_{{{\text{GL}}1}}$, $V_{{{\text{GL}}2}}$, and $V_{{{\text{GR}}1}}$ are floating. As $V_{{{\text{BG}}}}$ is increased, a large dip appears in $\left| {\Gamma } \right|$ around $V_{{{\text{BG}}}}$ = 2.5 V, implying electron accumulation in the reservoir. At $V_{{{\text{BG}}}}$ higher than 4 V, several small dips appear in the amplitude, corresponding to Coulomb peaks seen in (**b**). (**c**,**d**) Extracted device capacitance $C_{{{\text{dev}}}}$ and conductance $G_{{{\text{dev}}}}$ as a function of $V_{{{\text{BG}}}}$. These are obtained by subtracting the inductor and capacitance impedances with parasitic components from the measured load impedance.

Next, we investigate the reflection dependence on back gate voltage, $V_{{{\text{BG}}}}$, at a constant frequency, $f$ = 223.464 MHz which corresponds to the resonance frequency at $V_{{{\text{BG}}}}$ = 5.4 V. Figure 3a,b show the DC QD current, $I_{{{\text{QD}}}}$, and the amplitude of ${\Gamma }$, $\left| {\Gamma } \right|$, as a function of $V_{{{\text{BG}}}}$. Hereafter, the amplitude and the phase of ${\Gamma }$ as a function of a gate voltage are corrected in a similar manner described above by taking into account the external components. The QD device can also have parasitic components such as gate capacitance, $C_{{2{\text{D}}}}$, and its dielectric loss, $G_{{2{\text{D}}}}$. We derive the device admittance, $Y_{{{\text{dev}}}} { } = { }G_{{{\text{dev}}}} { } + { }i\omega C_{{{\text{dev}}}} ,$ where $G_{{{\text{dev}}}} = G_{{{\text{QD}}}} + G_{{2{\text{D}}}}$ and $C_{{{\text{dev}}}} = C_{{{\text{QD}}}} + C_{{2{\text{D}}}} ,$ from the $V_{{{\text{BG}}}}$ dependence by subtracting impedances of inductance and parasitic capacitance together with their additional parasitic components (Fig. 3c,d). As seen in Fig. 3c, $C_{{{\text{dev}}}}$ qualitatively agrees with a result in standard CV measurements of MOS capacitors^13^, which is reasonable because the only difference is the direction of signal: from gate to semiconductor (CV measurement of MOS capacitors) or from semiconductor to gate (RF reflectometry of QDs). The peak in $G_{{{\text{dev}}}}$ around $V_{{{\text{BG}}}}$ = 2.5 V is also similar to the one for CV measurements which can be explained by the effect of dielectric loss related to oxide (Fig. 3d)^30–32^. In addition to being useful for establishing a detailed device model, understanding this *in-situ* tunability can potentially provide an on-chip implementation of gate-tunable reflectometry circuits previously achieved via external components such as varactors^33–35^. At higher voltages, oscillations appear in $\left| {\Gamma } \right|$, corresponding to Coulomb peaks in $I_{{{\text{QD}}}}$, which implies the successful realization of RF-SET.

### Side gate voltage dependence

**Figure 4:**
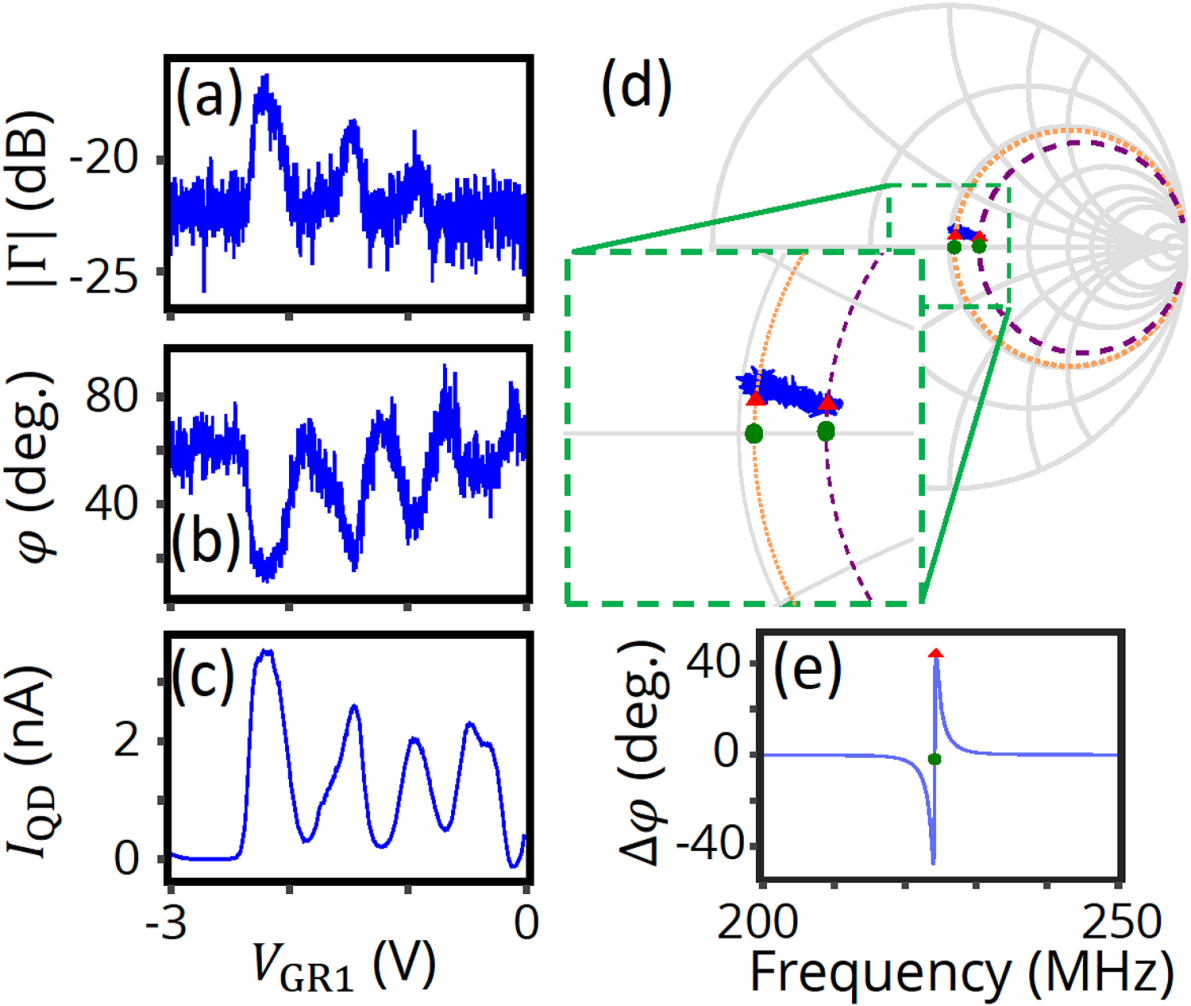
Side gate voltage of RF reflectometry and simulation with the equivalent circuit. (**a**–**c**) Amplitude and phase of ${\Gamma } = \left| {\Gamma } \right|{\text{e}}^{{{\text{i}}\varphi }}$, and $I_{{{\text{QD}}}}$ as a function of SG voltage, $V_{{{\text{GR}}1}}$, at liquid helium temperature. $V_{{{\text{BG}}}}$ = 6 V, $V_{{{\text{DS}}}}$ = 1 mV, $V_{{{\text{GL}}1}}$ = $V_{{{\text{GL}}2}}$ = 0 V, and $f$ = 224.3 MHz. (**d**) Smith chart for the ${\Gamma }$ dependence together with frequency dependences of simulated reflection coefficient using the equivalent circuit in Fig. 2c (orange dotted circle and purple dashed circle). For the simulations, $L$ = 680 nH, $\rho_{{\text{L}}}$ = 190 μΩ/$\sqrt {{\text{rad}}/{\text{s}}}$, $C_{{\text{L}}}$ = 350 fF, $C_{{\text{p}}} + C_{{{\text{dev}}}}$ = 391 fF, and ${\text{tan}}\delta$ = 0.00614. $G_{{{\text{dev}}}}$ is set to be 4.73 μS for the orange circle and 8.43 μS for the purple circle. Red triangles (green dots) indicate ${\Gamma }$ at $f$ = 223.464 MHz (224.157 MHz). Red triangles qualitatively reproduce the ${\Gamma }$ change by Coulomb peaks. (**e**) Frequency dependence of phase difference between the two device conductance values, ${\Delta }\varphi$. A red triangle and a green dot correspond to the two frequency values used in (**d**).

We can expect the conductance sensitivity to further increase, when a SG voltage is swept instead of a back gate to suppress these changes in the device admittance related to the turn-on process. To perform such an experiment, we use another QD device with a nominally identical design and set the charge sensor and one of the side gates to be floating to avoid unintended RF paths. The measured RF reflectometry signals as a function of a SG voltage, $V_{{{\text{GR}}1}}$ are shown in Fig. 4a,b. We find that peaks (dips) in amplitude (phase) of ${\Gamma }$ nicely reproduce the Coulomb peaks of $I_{{{\text{QD}}}}$ in Fig. 4c. Strikingly, the observed phase shift ($\sim 45^{ \circ }$) is significantly larger than those observed in other RF-SETs^14,36^.

## Discussion

To understand the origin of the large phase shifts, we simulate the $V_{{{\text{GR}}1}}$ dependence of the reflected signal. In Fig. 4d, the simulation results are plotted in a Smith chart (orange dotted circle and purple dashed circles) together with the $V_{{{\text{GR}}1}}$ dependence (blue solid line). For the one shown by the purple dashed circle, $G_{{{\text{QD}}}}$ is set to be higher than for the other one represented by the orange dotted circle by 3.7 μS, in order to approximately simulate the Coulomb peak conductance, with the other parameters obtained by fitting the frequency dependence. Both simulation results show constant resistance circles as expected from the equivalent circuit in Fig. 2c. Remarkably, the orange one passes through almost the center of the Smith chart, meaning that the load impedance at resonance is closely matched to $Z_{0}$. The results at given frequencies are indicated by the red triangles (223.464 MHz) and the green dots (224.157 MHz) in the Smith chart: the red ones are for the frequency used in the measurement (223.464 MHz) and the green ones for the resonance frequency for the orange circle condition (224.157 MHz). As expected, the red triangles agree with the $V_{{{\text{GR}}1}}$ dependence experimentally observed. We also calculate the frequency dependence of the phase difference ${\Delta }\varphi$ expected for the same (3.7 μS) conductance change in Fig. 4e, with a red triangle and a green dot highlighting the same two frequencies as in Fig. 4d. It turns out that there are two maximums in the absolute value of ${\Delta }\varphi$. We note that the resonance frequency (marked by the green dot) is located in between the two maximum points and has a small phase shift ($\sim 2^{ \circ }$). On the other hand, the frequency used in the measurement (the red triangle) is located close to one of the maximums, and the large phase shift ($\sim 45^{ \circ }$) agrees excellently with data. This large phase shift will not occur if the load impedance at resonance is away from impedance matching, where larger or smaller constant resistance circles would appear. Therefore, we conclude that a good impedance matching and a small frequency detuning from the resonance frequency are the necessary ingredients for the large phase shift caused by Coulomb oscillations. We note that this large phase shift is caused by a conductance change, rather than a change in quantum or tunnel capacitances, for which much smaller phase shifts are typically reported (e.g. Refs.^14,36^). Observation of reflectometry phase shift due to a conductance change is scarce. However, in theory, the phase change can be as large as 180° when the system passes exactly across the matching condition. To the best of our knowledge, our result is one of the closest to this ideal situation in the literature.

## Conclusion

In conclusion, we have fabricated PD-QDs in SOI without the top gate structure and performed RF-SET measurements at liquid helium temperature. Based on an equivalent circuit for load impedance, the parasitic circuit parameters are estimated from the frequency dependence of RF reflection coefficient. Our method will be useful when one needs to modify the RF reflectometry technique to apply to new device structures. Furthermore, we find huge phase shifts corresponding to Coulomb peaks (${\sim 45^{\circ}}$), as a result of the combination of a good impedance matching and a detuning from resonance frequency. This is confirmed by simulation using the equivalent circuit model. We believe that our results will be helpful in designing reflectometry circuits through simulation in order to improve the sensitivity and speed of spin qubit readout.

## Supplementary Information

Supplementary Information

## Data Availability

The datasets generated during and/or analysed during the current study are available from the corresponding author on reasonable request.
