# Supplementary material for: Radio-frequency single electron transistors in physically defined silicon quantum dots with a sensitive phase response
Source: Sci Rep. 2021 Mar 12;11:5863. doi: 10.1038/s41598-021-85231-4 (PMC7955042; doi:10.1038/s41598-021-85231-4)
Supplement: Supplementary file 1 — Supplementary Information [file 41598_2021_85231_MOESM1_ESM.docx]

Supporting information:

Radio-frequency single electron transistors in physically defined silicon quantum dots with a sensitive phase response

Raisei Mizokuchi^1^, Sinan Bugu^1^, Masaru Hirayama^1^, Jun Yoneda^2^, and Tetsuo Kodera^1, *^

1 Department of Electrical and Electronic Engineering, Tokyo Institute of Technology, Meguro, Tokyo 152-8552, Japan

2 Tokyo Tech Academy for Super Smart Society, Tokyo Institute of Technology, Meguro, Tokyo 152-8552, Japan

S1. Estimation of parasitic components of the inductor at liquid helium temperature

To analyze the RF-SET characteristics, we obtain parasitic components of nominally the same inductor as used in the main text separately and in advance. Values of such parasitic components at room temperature are available in the datasheet of the inductor, but they will change at cryogenic temperatures due, e.g., to a decrease in copper wire resistance and a change in the permeability of the core.

The inductor impedance including parasitic components, $Z_{L},$ are extracted from transmission measurement at 4 K using a nominally identical inductor to that used in the main text (LQW2BASR68J00L, Murata Electronics). To do this, we first solder the inductor on a PCB to make a transmission path through the inductor. Next, we connect the PCB to a vector network analyzer (VNA) and then measure the transmission from port 1 to port 2, $S_{21}$, with the inductor at helium liquid temperature (blue solid lines in Figs. S1(b) and (c)). As expected, $\left| S_{21} \right|$ shows a dip corresponding to self-resonance due to parasitic capacitance of the inductor.

To fit the results, $S_{21}$ is formulated as follows. The voltage at each point in Fig. S1(a) can be described by:

$$\begin{aligned} V_{A}=V_{i}+V_{r},\#\left( S1 \right) \end{aligned}$$

$$\begin{aligned} V_{B}={(1+\Gamma_{B})V}_{i}e^{-i\beta l},\#\left( S2 \right) \end{aligned}$$

$$\begin{aligned} V_{C}=V_{B}Z_{0}/\left( Z_{L}+Z_{0} \right),\#\left( S3 \right) \end{aligned}$$

$$\begin{aligned} V_{D}=V_{C}e^{-i\beta l^{'}},\#\left( S4 \right) \end{aligned}$$

where $V_{i}$ and $V_{r}$ are incident and reflected voltages at point A, $\Gamma_{B} = Z_{L}/(Z_{L} + 2Z_{0})$ is the reflection coefficient at point B seen from left-hand side, $Z_{0} = 50 \Omega$ is the characteristic impedance, $l$ ($l^{'}$) is the length of the coaxial cable between points A and B (C and D), and $\beta$ is the propagation constant without attenuation of the coaxial cables which is proportional to carrier frequency. In addition, it is assumed that reflection at each VNA port does not occur thanks to impedance matching. Thereby, the transmission coefficient $S_{21}$ is

$$\begin{aligned} S_{21}=\frac{V_{D}}{V_{i}}=\frac{2Z_{0}}{Z_{L}+2Z_{0}}e^{-i\beta{(l + l}^{'})}.\#\left( S5 \right) \end{aligned}$$

Using this equation, we perform a fitting with $Z_{L}$ including the parasitic capacitance $C_{L}$ and resistance $R_{L}=\rho_{L}\sqrt{\omega}$. In Figs. S1(b) and (c), the fitting results are shown, which agree well with the measurements (red dotted lines). Here, the inductance is fixed to the nominal value of the inductor. From the fitting, following parameters are obtained: $C_{L}$ = 0.30 pF, and $\rho_{L}=190 \mu\Omega/\sqrt{\mathrm{rad}/s}$. The estimated value of $\rho_{L}$ is utilized for the fitting in the main text while that of $C_{L}$ is not because it depends on soldering condition.

At frequencies higher than the self-resonance frequency, a deviation from the fitting in the transmission appears, which may be attributed to other parasitic components such as the inductance of bonding wires. Here, their contributions are ignored because they are effective outside the frequency range used in the main text.

S2. Fitting with an equivalent circuit without additional parasitic components

We also perform fitting of frequency dependence of the RF-SET without additional parasitic components (Fig. S2). The equivalent circuit consisting of the inductance $L$, capacitance $C$, and conductance $G$ is shown in Fig. S2 (a). In Figs. S2 (b) and (c), the amplitudes and the phases of the measured reflection coefficient are plotted, respectively, along with the simulated results. Here, the measurement results are the same as the ones in Fig. 2 in the main text. We fit the data without introducing the additional parasitic components (red dotted lines in Fig. S2(b) and (c)). The results agree well with the measurement result; however, (the value of) $L$ used for the fitting is 2.36 μH which is 3.5 times larger than the nominal inductance. This can be understood by the same mechanism as the self-resonance of the inductor due to its parasitic capacitance. As the frequency approaches the self-resonance, the inductance increases until the inductor starts to behave like a capacitor above the resonance frequency. This result indicates the necessity of introducing the additional parasitic components for the fitting.

For the completeness, we also simulate the characteristics with $L$ fixed to the nominal value, 680 nH (green dotted lines in Fig. S2(b) and (c)). $G$ and $C$ are estimated from the load impedance calculated from the reflection at the resonance and from the resonance frequency. The result apparently deviates from the measurement data, also indicating requirements for additional parasitic components for the fitting.

**Fig. S1.** (a) An equivalent circuit for transmission measurement of an inductor at helium liquid temperature. Parasitic components in the PCB are ignored because of their large impedance compared to characteristic impedance $Z_{0} = 50 \Omega$. Transmission through the inductor with $L$ = 680 nH was measured using vector network analyzer. (b,c) Amplitude and phase of the transmission through the inductor as a function of carrier frequency. Measurement results (blue solid lines) are fitted with an equation from the equivalent circuit model (red dotted lines).

**Fig. S2.** (a) An equivalent circuit without additional parasitic components for RF reflectometry. (b,c) Amplitude and phase of the reflection coefficients as a function of carrier frequency. Measurement results (blue solid line) which are also shown in Fig. 2 in the main text are fitted based on the equivalent circuit in (a) (red dotted line), yielding $L$ = 2.36 μH, $C$ = 214 fF, and $G$ = 3.43 μS. Green dotted line shows a simulation result for which $L$ is fixed to its nominal value, 680 nH, while the other parameters are obtained from the resonance frequency and the amplitude of reflection coefficient at resonance: $G$ = 40 μS and $C$ = 740 fF.
